# Menstruating while homeless: navigating access to products, spaces, and services

**DOI:** 10.1186/s12889-024-18379-z

**Published:** 2024-03-27

**Authors:** Andrea L. DeMaria, Rebecca Martinez, Emily Otten, Emma Schnolis, Sofia Hrubiak, Jaclyn Frank, Risa Cromer, Yumary Ruiz, Natalia M. Rodriguez

**Affiliations:** 1 Department of Public Health, Purdue University, West Lafayette, IN USA; 2 Department of Anthropology, Purdue University, West Lafayette, IN USA; 3 Department of Biological Sciences, Purdue University, West Lafayette, IN USA; 4 School of Health Sciences, Purdue University, West Lafayette, IN USA

**Keywords:** Menstruation, Homelessness, Public health, Menstrual products, Safe spaces, COVID-19, Qualitative research

## Abstract

**Background:**

People experiencing homelessness (PEH) in the United States face substantial challenges related to menstruation, exacerbated by the COVID-19 pandemic. Limited access to period products, heightened stigma, and gynecological challenges contribute to increased hardships for PEH, highlighting the need for improved services and policies to address period equity and overall well-being for this vulnerable population.

**Methods:**

We conducted semi-structured qualitative interviews with PEH (*n* = 12) and community healthcare and social service providers (e.g., case managers, shelter directors, community health workers, and nurses, *n* = 12) in Lafayette, Indiana, a city located between Indianapolis and Chicago in the United States. We used thematic analysis techniques for data analysis.

**Results:**

PEH’s limited access to products, services, and safe spaces hindered effective menstruation management within restrictive community contexts. Although community healthcare and service providers offered some support, complex interactions with the healthcare system, stigma, and limited access to spaces exacerbated barriers. The COVID-19 pandemic further intensified these difficulties by closing public spaces, worsening economic conditions, and straining service provider resources.

**Conclusions:**

Results highlight critical organizational and policy gaps in the United States for menstruation management resources and services, emphasizing the need for better integration into health and well-being programs for PEH. These insights will advance reproductive and public health research, shedding light on the disparities faced by PEH in managing menstruation in Indiana and contributing to the national discourse on addressing these barriers. Amid the complex landscape of public health, particularly during and after the pandemic, prioritizing menstrual health remains essential for all individuals’ overall well-being, including those experiencing homelessness.

## Background

In the United States, menstruating people experiencing homelessness (PEH) face significant menstruation-related challenges [1–9]. Nearly 40% of homeless individuals are women, including some, but not all, who menstruate [10]. At any given time, nearly 600,000 people around the country experiencing homelessness sleep in temporary shelters or on the street [11]. A point-in-time count in 2019 estimated nearly 5,500 individuals experiencing homelessness in Indiana, though that number is reasonably underestimated because of the difficulties in the counting process [11]. Evidence suggests that PEH in the United States during the COVID-19 pandemic endured greater hardship due to the sudden lack of pedestrian donations and the closure of many public restrooms, soup kitchens, and other resources they typically relied on to meet basic needs [12–17].

During the COVID-19 pandemic, menstruation management barriers have been amplified and largely ignored [18]. Irregular access to supplies, toilets, private and hygienic bathing spaces, and laundering services [19], along with pervasive menstrual stigma [5] and gynecological challenges [20], amplify the difficulties of managing menstruation for PEH [21–25]. Menstrual stigma encompasses societal taboos and negative perceptions surrounding menstruation, perpetuating shame and discrimination [26]. For PEH, addressing menstrual stigma is crucial as it intersects with challenges they face, such as access to menstrual products, services, and community programs, contributing to broader issues of marginalization [21, 27].

Despite reports of unmet needs and heightened stress and stigma, menstruating PEH are likely forced to cope with creative strategies that, if studied, could identify key shortcomings of existing programs and improve services for all PEH. According to journalistic reports, some coping strategies include rationing period products and supplies, using alternatives (e.g., toilet paper, socks), and making difficult decisions about spending limited resources on menstruation management or other essential needs, like food, shelter, and transportation [1–5, 8, 9]. While menstrual health [28] is a key factor of general health, healthcare and service providers infrequently and inconsistently address menstruation and period-related issues during encounters with menstruating people who are homeless [22, 23, 29, 30]. In the United States, the median age of menarche (i.e., first menstrual period) is 12.43 years [31], and the average age of menopause is 52 years [32], suggesting a significant portion of a menstruating person’s life is spent managing their period.

In the United States, lack of access to menstruation products, due to an inability to purchase or unavailability in a moment of need, can negatively impact individual health, such as infection, irritation, and stress [33]. Along with these barriers to product access, many menstrual products are subject to an additional sales tax, including in Indiana [34]. To mitigate the burdens menstruators bear, Purdue University [35, 36] and some communities—like West Lafayette, Indiana [37]—have implemented programs offering free menstruation products [38–40]. The primary facilities in Lafayette, Indiana that serve PEH are LTHC Homeless Services (LTHC) and Lafayette Urban Ministry (LUM) Emergency Shelter. LUM provides overnight shelter, meals, and showers whereas LTHC provides meals, case providers, laundering services, daytime shelter, case workers, menstruation products, clothing donations, and showers. The provision of free menstruation products is gaining support through state-level legislation, known as “period policies,” that strives to improve the safety and accessibility of menstruation products [41–43]. Free menstruation products may mitigate social stigma by providing a reliable process for menstruators to acquire the products they need without negative social consequences. However, many shelters are still functioning on a short-term approach to donations. Policies addressing the root causes, such as including menstrual products in federal assistance programs and mandating free products at educational institutions, could enhance period equity for PEH [44].

Menstruation-related challenges for PEH can lead to lasting impacts, including health risks, mental health struggles, social isolation, and hindered education and employment opportunities [45]. It is imperative to understand the menstrual health needs of PEH, and how the COVID-19 pandemic impacted these needs, to identify effective practices for reducing period product and health access disparities and stigma. Challenges in menstruation management while experiencing homelessness increase individuals’ vulnerability to gynecological infections [21], a problem exacerbated by the COVID-19 pandemic [46, 47]. PEH lack product access and essential supplies for menstruation [46, 48] and are more likely to experience COVID-related morbidities and mortalities [47]. Despite this, few studies have investigated menstruation experiences of PEH, resulting in a significant knowledge gap in the understanding and practices of PEH. With limited knowledge, policies and programs have improperly addressed the menstrual health needs of PEH, a population expected to surge due to COVID-19 [49, 50].

### Study purpose

Our study is among the first conducted in the Midwest, expanding upon previous research and investigations in NYC and St. Louis [21, 27, 46, 48, 51]. Our team built upon this preexisting research and leveraged an ongoing local community-academic partnership on homelessness and health [52] to further study the structural and social barriers faced by PEH [53, 54] and to better understand the specific barriers and menstruation management strategies of the Tippecanoe County homeless population. This research will inform future community-based interventions to improve menstrual health resources, policies, and protocols for PEH and other socioeconomically disadvantaged individuals.

This study sought to gather insights from PEH and service providers to inform policies and programs addressing menstrual needs among PEH. Our team focused on understanding PEH’s experiences and practices regarding menstruation. With additional input from service providers, our team could develop a better understanding of the current services and facilities available to PEH, as well as the struggles faced by service providers. Additionally, we explored how the COVID-19 pandemic impacted menstrual management for PEH and the support provided by community service providers. By comparing information gathered from PEH and service providers, our team will better inform policymakers and service providers on steps needed to improve the menstruation management experience for PEH.

## Methods

This research was part of a more extensive study investigating menstruation and reproductive healthcare experiences of PEH in Tippecanoe County, Indiana, to understand the specific barriers, perceptions, and practices of menstruators experiencing homelessness. In-depth interviews were conducted in English with two groups: menstruators experiencing homelessness (*n* = 12), and social service and healthcare providers (*n* = 12). Interviews were conducted privately, virtually or in person, between one female interviewer, and at times one female undergraduate researcher conducting field notes, and the participant. Interview guides were developed based on research on homelessness experiences to investigate the experiences of menstruators experiencing homelessness and the perceptions and knowledge of service providers who work with PEH. In addition to interviews, ethnographic methods were used, including participant observations of community health activities at the transitional housing center and informal interviews with community health workers and staff. The resulting field notes and data informed participant recruitment, interview guides, and analysis. An ethnographic approach grounded the qualitative interviews in observational data and informal interviews. Qualitative methods were utilized to provide an in-depth look at the thoughts and experiences of participants. Purdue University’s Institutional Review Board (IRB) approved the study. Before the interview, all participants, PEH and service providers, gave verbal informed consent. If an interviewee was emotionally overwhelmed or uncomfortable during the interview, they were offered time to compose themselves or stop the interview altogether. As compensation for their time and efforts, PEH were provided their preferred menstrual products (from a basket of offerings) and a gift card and service providers were provided solely a gift card.

Recruitment for participants began in July 2021 and ended in January 2022 when researchers agreed on achieving data saturation. Researchers recruited PEH by placing flyers at a homelessness service agency, the primary engagement center for PEH in Tippecanoe County, and through public announcements at the same center. PEH eligibility required participants to be at least 18 years old, assigned female at birth, have experienced menstruation since March 2020, and had received support for homelessness at the time of the study. Menstruators were asked about personal experiences with menstruation while homeless and before experiencing homelessness. By privately conducting interviews with PEH, researchers ensured any responses by PEH would not affect access to services or treatment within service centers.

Recruitment for service providers occurred through direct email. Providers known to offer support and health services to PEH were recruited using researcher professional networks, online profile information, and snowball sampling. All social service and healthcare providers confirmed engagement with PEH in Tippecanoe County and provided support, education, or community programming services. All participants completed an electronic consent form and demographics survey prior to interviewing. Service providers were asked about community resources for PEH, and their own experiences engaging with PEH.

Interviews followed a semi-structured protocol, which allowed researchers to alter or add questions to expand upon research topics discussed during the interview (see Table 1 for representative primary and probing questions). Interviews were recorded and transcribed using Otter.ai, and two team members reviewed transcripts to ensure accuracy. We utilized a thematic analysis [55] approach to data analysis, where each transcript was reviewed systematically, noting impressions of topics and emerging themes. These impressions and themes were used alongside the interview guides, ethnographic participant observations, and existing literature to combine inductive and deductive coding schemes. Researchers coded transcripts using NVivo 12, a qualitative data analysis computer software package, until saturation was reached (i.e., no additional new codes were added to the data set). Following the coding process, data were organized into themes and subthemes, with regular meetings held to discuss themes and findings.

**Table 1:** **Representative Interview Questions** Complete interview guides are available by request. Please email Andrea L. DeMaria at ademaria@purdue.edu

PEH QUESTIONS	SSP/HCP QUESTIONS
Thinking about your more recent periods, tell me about your experiences with them since coming to LTHC/when experiencing homelessness? How would you describe your current periods? (Flow, number of days, any pain or discomfort) And how do you feel about it? (normal, expected, not normal, unsure) What do you typically use when on your period? Where do you get these items? How easy is it to get what you need? What are those experiences like getting these things? Do you like using the products you use or is there something else you would prefer? How many tampons/pads/other period products do you need on your period? Do you have access to how much you need? Where do you go when needing to change your menstrual product or bathe during your period? How would you describe these places (e.g. safe, clean, private)? What has helped you deal with your period while experiencing homelessness (e.g. open facilities, products, programs, organizations, people)? What has made it more challenging? Can you explain why and how you have dealt with these challenges? What do you most need to manage your periods right now? (e.g., products, services, facilities, etcetera) I would like to ask you a bit about COVID-19. How has COVID-19 impacted you? There has been a local and national ban on evictions since the start of COVID-19, how has this impacted you? Has in changed your access to housing? How has COVID affected how you deal with your period? Made it easier? Made it harder? In the last 18 months, have you noticed any changes on your period? (increased or decreased flow, pain, discomfort) If you received the COVID-19 vaccine did you notice any differences in your period? Have you had to use different things for your period since the start of the pandemic? If yes, what and why was that? We’ve discussed where you go when needing to change your menstrual product or bathe during your period– how has this changed during the pandemic? What has made your period more challenging? Why is that? And how you have dealt with these challenges? What has helped you deal with your period during the pandemic (e.g. open facilities, products, programs, organizations, people)?	What spaces are available in your organization for menstruators to use to manage their menstruation? Such as toilets, showers, sinks, etc. How would you describe the condition of these spaces? Where else do you think people experiencing homeless go to manage their menstruation? Hygiene? Access menstrual products? Thinking about menstruation among people experiencing homelessness, from your perspective, what is that experience like for them? Has anyone ever shared with you their experiences? What did you learn from those experiences? What do you think are some of the challenges menstruators experiencing homelessness face when it comes to their menstrual health? How do the challenges surrounding menstrual health affect other aspects of their health? How do the challenges surrounding homelessness affect other aspects of their health? Have you noticed or are you aware of any differences between newly vs. longer-term/chronic homeless individuals in terms of menstruation management? Since the start of COVID-19, what challenges have you noticed about how people experiencing homelessness deal with menstruation and menstrual health? How have available supplies or spaces to change menstrual products changed? What have you/your organization done to address challenges? What are the largest challenges this population has faced? In what ways can service providers, like yourself, more effectively deal with the menstruation needs of people experiencing homelessness? How can the city more effectively support your clients with this need? [e.g. supplies, public toilets, disposal] What would you recommend to policymakers? If a training program was built to educate service providers on this topic, how do you think this should be delivered (e.g., online, in person, individually, group)? Should this be required? What would be helpful to you and your colleagues?

## Results

Participant quotes are noted below to illustrate our findings. Menstruator quotes are de-identified, while service provider quotes are followed by their professional title (i.e., [Health Care Provider (HCP)], [Social Services Provider (SSP)]). Participant characteristics are described in Table 2. Overall, three themes resulted from the data related to (1) menstrual symptoms and experiences, (2) menstrual product and public facility access, and (3) the impact of COVID-19 on menstruation experiences.

**Table 2:** Participant Characteristics

	PEH	Service Providers
**Age (years)**	38.75 ± 7.14	37.25 ± 12.83
**Gender Identity**		
Cisgender woman	11 (91.67%)	10 (83.33%)
Cisgender man	0 (0.00%)	2 (16.67%)
Gender non-conforming	1 (8.33%)	0 (0.00%)
**Sexual Orientation**		
Lesbian or Gay	2 (16.67%)	3 (25.00%)
Straight	7 (58.33%)	6 (50.00%)
Bisexual	1 (8.33%)	2 (16.67%)
Other	2 (16.67%)	1 (8.33%)
**Highest level of education obtained**		
Some high school or less	2 (16.67%)	N/A
High school diploma	7 (58.33%)	N/A
Vocational training or college degree	3 (25.00%)	N/A
**Marital Status**		
Single/Never married	3 (25.00%)	N/A
Separated	2 (16.67%)	N/A
Divorced	3 (25.00%)	N/A
Married/Partnered	4 (33.33%)	N/A
**Ethnicity**		
Not Hispanic or Latino	11 (91.67%)	10 (83.33%)
Hispanic/Latino	0 (0.00%)	2 (16.67%)
Unknown	1 (8.33%)	0 (0.00%)
**Race**		
Black or African American	1 (8.33%)	0 (0.00%)
White or Caucasian	8 (66.67%)	9 (75.00%)
Asian or Asian American	N/A	1 (8.33%)
More than one race	3 (25.00%)	2 (16.67%)

Note: Data presented as Mean ± SD or n(%). Numbers that do not add to 100% reflect missing data.

### “Because being homeless and being on your periods is…not comfortable. And it’s not safe”: Menstrual Symptoms

#### Physical Symptoms

Several participants noted they experienced a “really heavy” (PEH012) period and had symptoms including “cramping” (PEH011), “sharp pains” (PEH001), “tenderness of breasts” (PEH002), and “headaches” (PEH007). Participants explained many ways in which they managed their period-related symptoms. When discussing cramps, one participant expressed: “I need Tylenol, Advil, whatever I can get my hands on” (PEH004), and another stated: “I take Midol” (PEH011). When discussing challenges relating to menstruation while experiencing homelessness, PEH noted not having a comfortable place to find relief, “I wish I had a place to lay my head down” (PEH003).

Multiple service providers explained they face barriers when assisting PEH to access medication to relieve symptoms: “we’re not even allowed to dispense medicine” (SSP004), while another stated: “[the] front desk is not allowed to hand out any pain relief” (SSP009). Based on the ethnographic data and observations, medicine was unavailable for PEH unless prescribed by a clinician and filled at a local pharmacy. In addition to symptom and pain management, multiple participants expressed using a form of “birth control to help regulate” (PEH002) their period and symptoms. While some PEH mentioned using birth control to help regulate their periods, they may not always have access to it.

#### Feelings

A general sentiment voiced by many was the difficulty of being outside when menstruating. PEH expressed various feelings (e.g., frustration, vulnerability, embarrassment, and shame) associated with their period experiences. Specifically, several menstruators described feeling vulnerable when menstruating while homeless, with one participant describing:

…especially when you don’t have um, a crisis bed or you don’t have a shelter to go to. So, you’re laying outside in the grass on your period, bugs are going to be at you. People don’t realize that bugs, insects, animals, they’re attracted to blood (PEH001).

PEH described their period coinciding with personal frustration, with one participant stating: “I have to find anger management within myself, especially when I’m on my period I get cranky and I act real bitchy” (PEH 006).

With a lack of access to products and spaces for menstruation management, PEH are recurrently forced to disclose their period to others. PEH expressed feelings of shame and embarrassment when discussing approaching others for obtaining period products: “I know there’s places and you can go to ask them, or sometimes it comes down to it, you have to ask a complete stranger. And that might be embarrassing, but what can you do?” (PEH001). Service providers further affirmed the negative perception of menstruation when discussing how they personally would feel when disclosing their period to a stranger: “I would say that I would be a little embarrassed” (SSP001). With a general stigma surrounding menstruation, one social service provider sensed that asking male workers or providers for products is especially embarrassing for PEH: “She had to go ask him for products and I’m sure that was horribly just embarrassing and humiliating for her to have to do and she still had to do it” (SSP002). Some PEH expressed indifference when discussing feelings on periods, but many expressed feelings of embarrassment and shame.

### “You have to change your tampon before they close”: Public Access to Facilities and Products

PEH mentioned using a variety of public, private, and donated resources to manage their period. In particular, one mentioned how their shelter supported their menstruation needs:

Being homeless, I stay at the shelter. I can just do it at the shelter, [it] has a bathroom, a shower, and stuff like that. But if I’m out and about, usually there’s a restroom somewhere I can go, a public restroom (PEH004).

While, another PEH noted the products they received were “uck” and they preferred products that were “comfortable,” “concealable,” and “normal” (PEH003).

Service providers corroborate the support of shelters by explaining available supplies, with one mentioning, “You know, socks, underwear, [period] products, any kind of toiletries, they can get all of those from the front desk” (SSP001). However, providers noted the embarrassment of items being kept or guarded: “You shouldn’t have to go ask someone for permission to have menstrual products” (HCP002). Service providers highlighted the reliance of facilities and shelters on menstrual product donations, which significantly curtails the choices available to PEH for managing their menstruation. This results in limited product quantities, compromised quality, and a lack of choice in product features, ultimately constricting the menstruation management options for PEH.

Outside the shelter setting, several PEH expressed difficulties with accessing bathroom and cleaning facilities at night:

Some of these, [the] majority of these places aren’t open 24/7. So, you have to pee like at 11, 11:30 before they close and you have to change your tampon before they close, okay. I’ve seen some women peeing in alleys, okay, and like, not to be gross, but like changing their [period] products in alleys and throwing them in dumpsters (PEH011).

Another PEH echoed the difficulty of relying on public restrooms for menstruation management: “And when it comes to a period, and you don’t have clothes, those people are still gonna close… I can’t wash myself and wash my underwear” (PEH 002), noting the challenges of accessing wash facilities and supplies when needed. Some participants described using different things instead of period products when they could not access them. In particular, one participant stated: “Oh I’ve had to use, I mean, I’ve had to use wadded up toilet paper” (PEH003).

Service providers and PEH highlighted the limitations in accessing menstrual products, emphasizing the need for more autonomy and challenges in obtaining essential items. One service provider noted “SNAP doesn’t pay for [period] products, it doesn’t pay for toiletries, toilet paper, that kind of stuff so that’s a limitation” (SSP008).

### “With COVID, a lot of places don’t have public restrooms anymore”: Impact of COVID-19 on Menstruation Experiences

COVID-19 posed significant challenges to accessing products and private spaces. With shelter necessities (e.g., underwear, socks, toiletries) “mainly run by donations” (SSP009), providers had varying perspectives on how COVID-19 impacted donations. One service provider stated: “People didn’t wanna spend the extra money to donate” (HCP002), while another service provider stated: “We’ve actually gotten more donations since then, because people are like, how can we help” (SSP004). Alongside unpredictable fluctuations in donations during COVID-19, service providers found other avenues for obtaining products. One service provider explained: “They didn’t offer those [pads and tampons] anymore as part of COVID. And so, the Lafayette Meijers, both Lafayette and West Lafayette Meijers, donated all of [the pads and tampons we supplied] to us” (SSP002). In addition to relying on donations, service providers discussed the difficulties of purchasing period products, stating: “The price of the products just keeps going up since COVID” (HCP002).

Many PEH expressed difficulties navigating public restrooms when looking for facilities to manage menstruation. In particular, one participant stated: “With COVID, a lot of places don’t have the public restrooms anymore, or you can’t get into a building because of COVID” (PEH003). Service providers observed similar impacts related to the issue of the shutdown of public spaces due to COVID, stating: “One of the big things was that whenever there was a positive case of COVID in the shelter, it would shut down and not accept new people” (HCP002). Another service provider explained: “So when they have to shut down, that go, that all their s-, all their free meals goes away, ability to wash their clothes, shower, receive their mail all that goes away. So that, I mean what, so you, they literally have nothing. Nothing” (SSP006). With facilities (i.e., shelters and public restrooms) shutting down due to COVID protocols, service providers struggle to provide resources and safe sleeping spaces, which PEH told us are fundamental to managing menstruation.

## Discussion

Menstruators experiencing homelessness face significant challenges and barriers to their menstrual health. Individuals navigated restrictive community resources with limited access to products, services, and spaces to manage their menstruation while homeless. While community healthcare and service providers offered some connection to spaces, products and services, menstruators experienced complex interactions with these providers and the healthcare system, exacerbated by social stigma and limited healthcare access. The COVID-19 pandemic further magnified healthcare access barriers and disparities for menstruators experiencing homelessness as public spaces closed, economic conditions deteriorated, and health outcomes were poor among those most socioeconomically disadvantaged. This research has illuminated the multifaceted challenges faced by PEH in managing menstruation. It has underscored the urgency of addressing this issue through a comprehensive approach, including policy and advocacy, community outreach and collaboration, education and awareness, support services integration, and ongoing research. Many participants described frequent menstruation-related pain, and heavy periods, leading to difficulties when managing menses. These concerns highlight a need for more access to clinical care services to identify causes of pain and heavy bleeding and social services to provide access to diverse products and infrastructure such as bathrooms and rest areas.

Period product access among PEH is a critical public health concern with far-reaching implications. The intersection of menstrual stigma and homelessness stigma can intensify challenges for individuals, leading to restricted access to menstrual products and services within community programs. This compound stigma not only reinforces systemic marginalization but also contributes to a cycle of increased difficulties in obtaining essential resources and fair treatment, creating additional barriers for those experiencing homelessness [26, 46, 56]. Findings demonstrate that a lack of access to menstrual products profoundly affects physical and mental well-being. Service providers described difficulties (e.g., guarded supplies, limitations of government programs/funds) when providing symptom and pain management medications and products, which exacerbate the challenges PEH experience when searching for necessary period products. When products were accessible, such as in shelters, participants frequently noted emotions of shame or embarrassment when requesting access to menstruation products. By adapting social service policies and increasing access to products and primary care for this population, many issues relating to obtaining over-the-counter medication for pain management and period products can alleviate PEH’s burden when managing their symptoms and menstruation. However, more research is needed as to the specific barriers, whether that be policies, stigma, or availability of medication, to pain management within homeless service providers. This issues of supplies corroborated in investigations in other cities [20, 21, 57, 58], indicating the menstruation experience of PEH as a whole is complicated by inadequate access to necessary items and services.

The scarcity of accessible, hygienic restroom facilities for changing menstrual products poses a notable challenge and hardship for PEH. Public restrooms are often limited in number and are frequently considered unclean or unsafe by PEH. Furthermore, the closure of facilities during certain hours compounds the issue, hindering timely access to essential facilities. Due to restricted bathroom availability, some PEH resort to makeshift solutions, such as wadded toilet paper or secluded areas like alleys. Adequate access to essential menstrual products, such as pads and tampons, clean restroom facilities, and showers, emerges as a paramount challenge in managing menstrual health for PEH [27]. Product donations should consider PEH preferences, such as absorbency, size, and comfort, until more cities and states fund these in shelters [46].

The COVID-19 pandemic posed formidable challenges for PEH and service providers, particularly impacting menstrual health and access to essential resources. Service providers faced unpredictability in obtaining menstrual products due to donation fluctuations, prompting them to explore alternative resource channels. Concurrently, PEH encountered difficulties accessing public restrooms as closures and limited hours exacerbated their struggles in finding suitable spaces for menstruation management. These findings align with a study in New York [21], underscoring the systemic issue of insufficient facilities and resources for menstruators experiencing homelessness. In response, urgent and sustainable solutions are needed to ensure consistent access to menstrual products and hygienic facilities, particularly during crises like the COVID-19 pandemic.

### Implications

Centering marginalized people in research that supports structural changes responsive to their needs and ideas often benefits all groups [59, 60]. Informed by this approach, our study centers on the experiences and insights of menstruating PEH to characterize barriers this population faces that may inform service and policy interventions. Our study advances reproductive and public health research goals by illuminating PEH’s challenges when managing menstruation in Indiana. It contributes to the national dialogue on the importance of addressing these barriers, through increasing product and hygienic space access [21, 48, 58], changing policies [34, 61], and improving period equity [62]. Additionally, our study draws attention to critical gaps at organizational and policy levels in menstruation-related services to better integrate menstruation into health and well-being programs for PEH.

This research underscores the broader implications of menstrual product access as a reflection of larger systemic issues such as poverty, gender inequality, and inadequate social support structures. Addressing the menstruation needs of PEH is not just a matter of individual health; it is a critical step toward promoting human rights, fostering inclusivity, and dismantling the social structures (i.e., economic inequality, lack of affordable housing, systemic discrimination, educational and healthcare disparities, substance use challenges, trauma and mental health issues) that perpetuate homelessness. Concerted efforts are needed to provide consistent and free access to menstrual products through shelters, outreach programs, and community initiatives. Policy changes and advocacy are imperative to effect lasting change and can be informed by these findings, such as including menstrual products in federal assistance programs, mandating free products at educational institutions, training healthcare and service providers on addressing the menstruation needs of PEH, and altering intake questionnaires to identify menstruation-related needs and challenges. Taking seriously the understanding of the pain of menstruators and PEH is something that demands more attention. By engaging with stakeholders across various sectors (e.g., public health, social services, healthcare providers, and policymakers) a collaborative approach can be established to ensure that the menstruation needs of PEH are not overlooked.

### Strengths & limitations

Our study employed qualitative research methods to understand the experiences of PEH and service providers, mainly focusing on the intersection of homelessness and menstruation. It is essential to consider the limitations of this study within this context. While our sample size adhered to qualitative research guidelines [63] (*n* = 12 PEH; *n* = 12 HCP/SSP), it may have constrained the diversity of experiences shared with the researchers. All interviews were conducted in English, potentially excluding the perspectives and experiences of individuals who may not be proficient in the language. Additionally, challenges arose during the recruitment of healthcare providers, specifically those working in certain facilities within the community, thus limiting the scope of the investigation to some but not all homeless-serving institutions in Lafayette, Indiana. Despite these recruitment difficulties, a range of healthcare and social service providers were interviewed, providing a comprehensive understanding of their perspectives. Our use of a semi-structured interview method introduced slight variations in interview topics, enriching the overall understanding of themes related to menstruation and menstrual health in the context of homelessness. This approach allowed for dynamic and comprehensive interviews, ensuring diverse viewpoints and experiences were captured from both PEH and service providers. Our findings support research conducted in large metropolitan communities (NYC [21] and St. Louis [48]) and extend the discourse by showcasing results from the small urban centers in Indiana.

## Conclusions

Our findings showcase the challenges faced by menstruators experiencing homelessness in Indiana and catalyze broader societal change. We advocate for integrating menstrual health into comprehensive public health and well-being programs, emphasizing the urgent need to address access barriers among PEH. By contributing to the national dialogue on reproductive and public health, our study aims to foster inclusivity and drive structural changes responsive to the needs of marginalized communities. Additionally, our research underscores the significance of targeted interventions and policies while advocating for a paradigm shift towards human rights, inclusivity, and dismantling systemic issues perpetuating homelessness. We call for a collective commitment to action, urging stakeholders across sectors to collaborate in implementing tangible interventions and policies that integrate menstrual health into public health programs. This proactive approach is essential for addressing the urgent needs of menstruators experiencing homelessness and instigating lasting societal change among PEH.

## Data Availability

Data, coding schemes, and interview guides are available by request. Please email Andrea L. DeMaria at ademaria@purdue.edu.
